# Anti-Platelet Factor 4/Heparin Antibody Formation Occurs Endogenously and at Unexpected High Frequency in Polycythemia Vera

**DOI:** 10.1155/2017/9876819

**Published:** 2017-06-18

**Authors:** Sara C. Meyer, Eva Steinmann, Thomas Lehmann, Patricia Muesser, Jakob R. Passweg, Radek C. Skoda, Dimitrios A. Tsakiris

**Affiliations:** ^1^Diagnostic Hematology, Department of Laboratory Medicine, University Hospital Basel, Basel, Switzerland; ^2^Division of Hematology, Department of Medicine, University Hospital Basel, Basel, Switzerland; ^3^Experimental Hematology, Department of Biomedicine, University Hospital Basel, Basel, Switzerland

## Abstract

**Background:**

Myeloproliferative neoplasms (MPN) encounter thromboses due to multiple known risk factors. Heparin-induced thrombocytopenia (HIT) is a thrombotic syndrome mediated by anti-platelet factor 4 (PF4)/heparin antibodies with undetermined significance for thrombosis in MPN. We hypothesized that anti-PF4/heparin Ab might occur in MPN and promote thrombosis.

**Methods:**

Anti-PF4/heparin antibodies were analyzed in 127 MPN patients including 76 PV and 51 ET. Screening, validation testing, and isotype testing of anti-PF4/heparin Ab were correlated with disease characteristics.

**Results:**

Anti-PF4/heparin antibodies were detected in 21% of PV and 12% of ET versus 0.3–3% in heparin-exposed patients. Validation testing confirmed anti-PF4/heparin immunoglobulins in 15% of PV and 10% of ET. Isotype testing detected 9.2% IgG and 5.3% IgM in PV and exclusively IgM in ET. IgG-positive PV patients encountered thromboses in 57.1% suggesting anti-PF4/heparin IgG may contribute to higher risk for thrombosis in MPN. Overall, 45% of PV patients experienced thromboses with 11.8% positive for anti-PF4/heparin IgG versus 7.1% in PV without thrombosis.

**Conclusion:**

Anti-PF4/heparin antibodies occur endogenously and more frequently in MPN than upon heparin exposure. Thrombotic risk increases in anti-PF4/heparin IgG-positive PV reflecting potential implications and calling for larger, confirmatory cohorts. Anti-PF4/heparin IgG should be assessed upon thrombosis in PV to facilitate avoidance of heparin in anti-PF4/heparin IgG-positive PV.

## 1. Introduction

Myeloproliferative neoplasms (MPN) are clonal disorders of hematopoiesis with excessive proliferation of mature myeloid cells. They comprise several related entities [1] including chronic myeloid leukemia (CML) characterized by translocation t(9;22) known as the Philadelphia chromosome and the classical, Philadelphia-negative MPN. These present as essential thrombocythemia (ET) characterized by thrombocytosis, polycythemia vera (PV) with predominant erythrocytosis and concomitant leuko- and thrombocytosis, or myelofibrosis (MF) with expansion of megakaryocytes and reactive bone marrow fibrosis [2]. More rarely, chronic neutrophilic leukemia (CNL) or chronic eosinophilic leukemia (CEL) are also seen [1]. PV, ET, and MF represent the focus of this study and are subsequently referred to as MPN. They are characterized by hyperactive signaling of the JAK2 kinase [3, 4] essentially involved in hematopoiesis [5] which is constitutively activated by acquired gain of function mutations. These driver mutations affect* JAK2* itself, such as* JAK2*V617F in 95% of PV and 50% of ET and MF [6–9] and* JAK2* exon 12 mutations in PV [10]. In addition, mutations in the thrombopoietin receptor* MPL* [11], such as* MPL*W515L, or in the chaperone protein calreticulin* (CALR)* were identified in ET and MF, which converge on activation of JAK2 signaling [12–15].

While MPN potentially transform to acute myeloid leukemia with dismal prognosis [16], the most frequent complications in MPN are thrombohemorrhagic events [17, 18]. They relevantly contribute to the substantial disease burden and their avoidance is a major goal of current therapies. Thrombotic events exceed the hemorrhagic complications in MPN which occur mainly due to depletion of ultralarge von Willebrand factor (VWF) multimers by thrombocytosis leading to acquired von Willebrand syndrome (VWS) and altered platelet function [17, 19]. Thromboses in MPN affect both the arterial and the venous vascular beds comprising stroke or transient ischemic attacks, cardiac events, deep vein thrombosis, and pulmonary embolism as well as peripheral arterial or venous thrombosis [20]. Thromboses in MPN frequently present in atypical locations such as the splanchnic veins including mesenteric, splenic, portal, and hepatic vein thrombosis (Budd-Chiari syndrome) or cerebral venous sinuses. Thrombotic risk is highest in PV with 16–27% of patients affected by arterial and 7.4–11% by venous events [21, 22] and in ET with 10–50% of patients affected by a thrombotic complication within a decade from diagnosis [23]. Several risk factors have been identified and have been implemented in prognostic scores estimating thrombotic risk. While age over 60 years and history of thromboses represent the strongest predictors for thrombotic events [22, 24–30], the implication of leukocytosis, elevated hematocrit, and the* JAK2*V617F driver mutation have also been established. In addition, common cardiovascular risk factors as well as hereditary thrombophilia are considered relevant for overall risk of thromboses in MPN [17]. The pathogenesis of thromboses in MPN is currently perceived as multifactorial since general and disease-specific prothrombotic factors coincide. Thrombocytosis which is a characteristic presenting feature in ET, PV, and prefibrotic forms of MF has not been validated as a thrombotic risk factor in MPN.

The panoply of factors currently known to contribute to thrombotic risk in MPN might not be exhaustive and additional effects should be considered. Heparin-induced thrombocytopenia (HIT) represents a rare thrombotic syndrome mediated by an immune response to platelet factor 4 (PF4) secreted from platelet alpha granules [31]. PF4 complexes with heparin when the latter is administered for treatment or prophylaxis of thrombosis, giving rise to an immunogenic neoantigen. Consecutive antibody formation against PF4/heparin complexes is seen in 0.3–3% of patients on heparin treatment [32] and mediates activation and clearance of platelets leading to thrombocytopenia and potentially thrombosis, the full manifestation of HIT. Anti-PF4/heparin immunoglobulin production ceases upon withdrawal of heparin and anti-PF4/heparin antibodies subsequently become undetectable after heparin treatment is stopped. Even after overt HIT, anti-PF4/heparin antibodies are undetectable by 50–80 days [33]. Differential effects of several types of heparin are known such as increased risk of anti-PF4/heparin antibody formation by unfractionated heparin (UFH) as compared to low molecular weight heparin (LMWH) [34]. In addition, increased levels of PF4 in settings of platelet activation or high turnover have been shown to promote anti-PF4/heparin antibody formation probably via increased abundance of antigen [31]. Inflammatory stimuli by bacterial infection [35, 36] or by tissue damage upon surgery or major trauma are also considered promoting development of HIT [37, 38]. Interestingly, a recent study in orthopedic patients after knee or hip arthroplasty reported anti-PF4/heparin antibody formation in the absence of heparin treatment which was enhanced by dynamic versus static compression therapy for thromboprophylaxis [39, 40].

Insight into the significance of HIT for thrombosis in MPN is limited. The risk for thrombotic complications in MPN patients entails a high probability of heparin exposure during the course of the disease which could thereby put MPN patients at increased risk of developing HIT. However, diagnosis of HIT which includes substantial thrombocytopenia or a 50% fall in platelet counts is impeded by elevated baseline platelet levels in PV and ET. Thrombocytosis in MPN might mask occurrence of thrombocytopenia due to development of HIT, thereby leading to false negative assessments. Importantly, thrombocytosis in PV and ET is reflective of excessive platelet production and turnover, and platelets in MPN are known to circulate in an activated state, thus providing ample PF4 which could promote the formation of anti-PF4/heparin antibodies in PV and ET [17]. A limited number of PV and ET patients with occurrence of HIT have been reported (Table 1) [41–53]. In addition, analysis of a cohort of HIT patients found overrepresentation of ET and PV with 4.7% (2/42) [54]. The true incidence of HIT in PV and ET has not been clearly assessed given the masking of thrombocytopenia by excessive platelet production and the scarcity of the literature on this topic limited to a handful of case reports and series. A comprehensive case series studying 29 MPN patients [41] observed strong clinical evidence for HIT in five patients, but only two were tested for anti-PF4/heparin antibodies. Therefore, we employed a systematic approach to assess PF4/heparin antibody formation and its significance for thrombosis in a large cohort of PV and ET patients given their high thrombotic risk. We hypothesized that anti-PF4/heparin antibodies could be prevalent in these patients with excessive platelet production and turnover and might contribute to thrombotic risk in MPN.

## 2. Materials and Methods

### 2.1. Characterization of Patients

A cohort of 127 MPN patients including 76 PV and 51 ET patients from our tertiary care center diagnosed according to the WHO classification were retrospectively analyzed for MPN disease characteristics. Blood counts including hematocrit, hemoglobin concentration, platelet counts, white blood cell (WBC), and neutrophil counts were assessed on an ADVIA hemocytometer. The* JAK2*V617F mutation was assessed by allele-specific PCR in peripheral blood granulocyte DNA. Patient histories were assessed for occurrence, number, and localization of thrombotic events which were diagnosed by duplex sonography for deep vein thrombosis or CT angiography for pulmonary embolism and arterial events. Splenomegaly was assessed by clinical examination, sonography, or CT scan. Informed consent was available from all individuals and approval from the local ethics committee was obtained.

### 2.2. Anti-PF4 Antibody Testing

Testing for anti-PF4 immunoglobulins was performed in PV and ET patient serum samples stored at −80°C. We employed a sequential approach including a screening and a subsequent validation assay in positive samples for optimized specificity. Initial screening for anti-PF4 antibodies was performed by a commercial anti-PF4 ELISA globally detecting IgG, IgA, and IgM antibodies (ZYMUTEST HIA IgGAM ELISA, product number RK040D). Positive samples were subsequently subjected to specific isotype testing for validation and for determination of IgG, IgM, and IgA isotypes (ZYMUTEST HIA IgG, IgA, IgM, product number RK040E). IgG subclasses and FcgRIIa H131R polymorphism were not specifically assessed in the study.

### 2.3. Statistical Analysis

Statistical analysis was performed by SPSS software. Pearson's *X*2 test (two-by-two table) was used to compare categorical variables. Significance level was set at *p* < 0.05 in two-sided tests.

## 3. Results

### 3.1. Baseline Characterization of MPN Patient Cohort

The study population of 127 individuals consisted of 76 PV and 51 ET patients. Baseline characteristics are shown in Table 2. Among PV patients, there was a slight male predominance (*n* = 42 male, *n* = 34 female), while ET affected more women (*n* = 18 male, *n* = 33 female). Mean age at diagnosis was 55.8 years in PV and 53.2 years in ET. PV patients were characterized by erythrocytosis reflected by increased red cell parameters (mean hematocrit 53.2%, mean hemoglobin 176.2 g/l) as well as mild leukocytosis with mean white blood cell count (WBC) of 12.4 G/l. Red cell parameters and WBC were within normal range in ET patients, who displayed pronounced thrombocytosis with mean platelet count of 984.3 G/l. Thrombocytosis was also present in PV (mean platelet count 602.9 G/l).* JAK2*V617F was detected in 84.2% of PV and 52.9% of ET patients concordant with previous studies [6–9]. Splenomegaly by clinical assessment or imaging was more prevalent in PV (55.3%) than ET (41.2%). Thrombohemorrhagic complications were frequent with 63.1% in PV and 43.1% in ET. Thereof, 18.4% and 3.9% of patients suffered from bleeding events, respectively, while thromboses clearly outweighed bleeding events both in PV and in ET. Nearly all patients were on antiaggregatory prophylaxis with low dose aspirin (94.7% in PV, 88.2% in ET), while much less individuals had received anticoagulation with vitamin K antagonists during the course of their disease (30.3% in PV, 11.8% in ET). Substantial proportions had received cytoreductive therapy with 75.0% in PV and 68.6% in ET patients (Table 2). These analyses match well with established disease characteristics in PV and ET [2] and demonstrate that this patient population is representative and well suitable for investigations into the role of anti-PF4 immune responses in MPN.

### 3.2. Thromboses Are Frequent in PV and ET and Relate to Multiple Risk Factors

Thromboses occurred in 34/76 PV patients (44.7%) and 20/51 ET patients (39.2%) during the course of disease highlighting the very substantial contribution of thrombotic events to disease burden in MPN. The slightly higher incidence of thrombotic complications in PV as compared to ET patients is in accordance with previous reports [20, 21] (Table 2). In the presented patient population, cerebrovascular events were the most prevalent manifestation of thrombosis in 37.0% of patients, followed by venous thromboembolism in 31.5% including 20.4% of deep vein thromboses, 11.1% of pulmonary embolism, and 14.8% with cardial ischemic events (Table 3). Splanchnic thromboses which are characteristic for MPN were seen in 7/54 (13.0%) patients which represents a high incidence given their overall rarity. Two patients (3.7%) with peripheral arterial disease and additional vascular risk factors such as previous cigarette smoking, arterial hypertension, and dyslipidemia showed peripheral arterial occlusions. A substantial proportion (19/54 patients) suffered from repeated thrombotic events with 3 patients even showing multiple (3 and more) thromboses during the disease course. Platelet counts >400 G/l did not significantly affect thrombotic risk (*p* = 0.106), whereas patients with hematocrit >0.46 showed a significantly increased incidence of thromboses (*p* = 0.010), as did patients with leukocytosis >10 G/l (*p* = 0.033) reflecting established risk factors [17].

### 3.3. Anti-PF4/Heparin IgG Antibodies Occur at High Frequency in PV Patients

**Figure 1:**
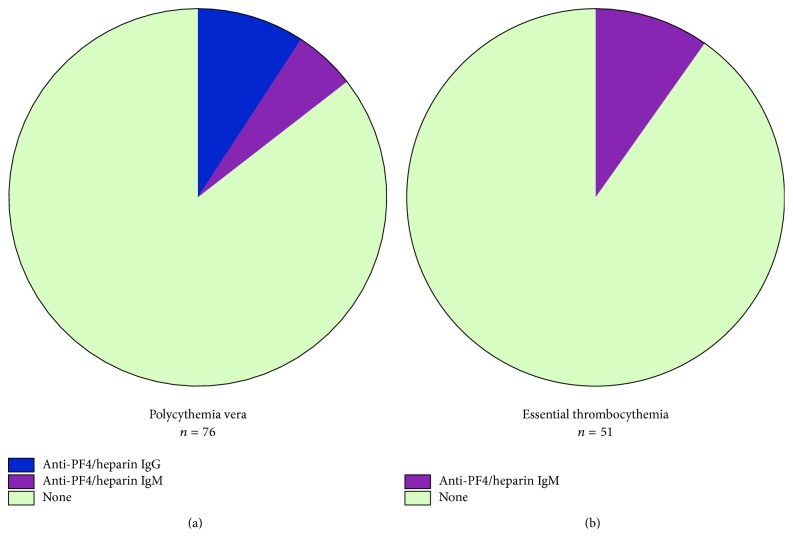
Endogenous anti-PF4/heparin IgG and IgM antibodies occur in polycythemia vera and essential thrombocythemia. (a) Anti-PF4/heparin antibodies of IgG isotype are detected at considerable frequency in polycythemia vera (PV), while IgM isotype antibodies are also found. (b) Anti-PF4/heparin antibodies in essential thrombocythemia are of IgM isotype in our cohort. No IgA isotypes were detected. PF4: platelet factor 4; Ig: immunoglobulin.

Qualitative screening for anti-PF4/heparin antibodies was positive in 22 individuals (17.3%) of the entire cohort of 127 patients. Incidence in PV patients was higher than in ET with 21.0% tested positive in PV and 11.8% among ET patients (Table 4). Positive patients underwent subsequent validation testing, which confirmed anti-PF4/heparin antibodies in 14.5% of PV and 9.8% of ET patients (Figure 1). Immunoglobulin isotype testing showed that 9.2% of PV patients had circulating IgG against PF4, for which a functional role as platelet activators in pathogenesis of HIT has been demonstrated [31]. The prevalence of anti-PF4/heparin IgG among PV patients in our cohort is clearly higher than in patients treated with unfractionated heparin, for whom anti-PF4/heparin IgG have been reported in 0.3–3% after a treatment duration of more than four days [32] or at lower frequencies when LMWH is used [34]. Sequential antibody testing and isotype testing in 3/7 IgG-positive PV patients revealed persistent positivity after 847, 674, and 182 days, respectively, which supports the notion that anti-PF4/heparin IgG immunoglobulins can occur endogenously in PV patients and that antibody formation can be maintained despite the absence of concurrent heparin exposure. Immunoglobulin isotype testing also identified anti-PF4/heparin IgM in 5.3% of PV patients. A thrombogenic potential of anti-PF4/heparin IgM has not been clearly established and a causative implication for thrombosis in HIT remains on debate. All anti-PF4/heparin antibodies detected in ET patients were of IgM isotype at a prevalence of 9.8%. No anti-PF4/heparin IgG were observed in ET patients in our cohort. As patients with ET showed more pronounced thrombocytosis than PV patients, the absence of IgG isotypes in ET suggests that excessive platelet production associated with increased circulating levels of PF4 may not be the sole factor facilitating anti-PF4 immune responses in MPN but that additional promoting factors are at play specifically in PV patients (Table 4). However, it is noteworthy that anti-PF4/heparin IgM antibodies persisted in a majority (5/9) of positive patients with 3/4 PV and 2/5 ET patients showing detectable IgM at two sequential assessments with intervals of 1003, 904, 811, 539, and 42 days. As several cases of clinically manifest HIT in ET patients have been reported (Table 1) and exclusively anti-PF4/heparin IgM were detectable in ET patients of our cohort, a potential implication of anti-PF4/heparin IgM for platelet activation in the setting of MPN with thrombocytosis may warrant further evaluation in functional studies.

### 3.4. Anti-PF4/Heparin IgG Antibodies Confer a Tendency for Increased Thrombotic Risk in PV

We next assessed to what extent anti-PF4/heparin IgG immunoglobulins with the known potential to activate platelets in the pathogenesis of clinical HIT would contribute to thrombotic complications in PV. We observed that 57.1% of PV patients with circulating anti-PF4 IgG suffered from a thrombotic event at least once during the course of disease (Table 5) including both arterial and venous events according to the known spectrum of thromboembolic complications in MPN (Table 6). Anti-PF4/heparin IgG-negative PV patients encountered thromboses in 43.5%, reflecting a 31% increase in relative thrombotic risk for PV patients with anti-PF4/heparin IgG as compared to IgG-negative PV in our cohort (Table 5, *p* > 0.05). Predictors of whether IgG-positive PV patients would actually develop thrombosis were absent suggesting that thrombosis in PV is a strongly multifactorial process, while the Fc*γ*RIIa H131R polymorphism was not assessed in this study. When PV patients with a positive history of thrombosis were assessed for anti-PF4 immune responses, we observed that 11.8% had anti-PF4/heparin IgG antibodies, as compared to 7.1% IgG positivity in PV patients who never had a thrombosis. Although the increased risk for thrombotic events in PV patients with anti-PF4/heparin IgG is statistically nonsignificant in our cohort, a subtle but relevant contribution of anti-PF4/heparin IgG to thrombosis in PV cannot be excluded at this point, particularly in view of the multifactorial nature of thrombosis in MPN with hematocrit and leukocyte levels as major risk factors. Further studies of more extensive patient populations are warranted to confirm the tendency for increased thrombotic risk in PV patients with anti-PF4/heparin IgG antibodies which we observe in our study. However, as PV is a rare hematological disorder, this will eventually require multicenter collaborative efforts.

Overall, we find a high frequency of circulating anti-PF4/heparin IgG antibodies in patients with PV which are detectable in the absence of concurrent exposure to heparin. There is a tendency for increased thrombotic complications in PV patients positive for anti-PF4/heparin IgG. Due to the retrospective nature of this study, data on antibody positivity at the time of thrombosis is not available for our cohort. However, our data suggest that PV patients should undergo anti-PF4/heparin IgG testing as soon as a thrombotic event occurs to prevent potential boosting of the anti-PF4/heparin immune response by initiation of heparin treatment as seen in rare reported cases [55] and to facilitate alternative anticoagulation in IgG-positive PV patients.

## 4. Discussion

We report on the prevalence of anti-PF4/heparin antibodies in a large cohort of PV and ET patients and evaluate to what extent anti-PF4/heparin IgG known to mediate platelet activation and consequent thrombosis in HIT would contribute to thrombosis in MPN. MPN are characterized by an acquired prothrombotic condition with multifaceted pathogenesis of interplaying proaggregatory and procoagulatory factors [17]. As a consequence, thrombotic events are frequent in MPN, particularly in PV. Clinically relevant thrombosis is found at time of diagnosis in 11–39% of PV and in 8–29% of ET [56, 57]. Thromboses often also complicate the further course of MPN as seen in 8–19% of PV and 8–31% of ET patients and outweigh the risk of bleeding complications, which also typically occur in MPN patients, but at lower frequencies than thromboses [19]. Thus, thromboses are a substantial contributor to symptom burden of MPN patients and impact on their life expectancies [58] as demonstrated by the largest epidemiologic study in PV (European Collaboration on Low dose Aspirin in Polycythemia vera, ECLAP), which showed cardiovascular events to account for 41% of all deaths [59]. The arterial circulation is more prone to thrombosis in MPN accounting for 60–70% of events mostly as cerebro- or cardiovascular complications and less frequently as peripheral arterial occlusions. Venous thromboembolism accounts for the remainder with a high incidence of thrombosis in atypical locations such as cerebral venous sinuses and splanchnic veins including mesenteric, portal, and hepatic vein thrombosis (Budd-Chiari syndrome). As MPN represent the most common cause of splanchnic vein thromboses, such events should always raise a high suspicion of underlying MPN [17].

As thrombosis related to HIT has been reported in patients with PV or ET (Table 1), we hypothesized that an immune response to PF4 may be relevant in MPN with a potential implication in thrombotic events. Strong predictors of thrombotic complications in MPN have long been known as a history of previous thrombosis and advanced age >60 years [22, 24, 26–30]. General risk factors for arterial or venous thrombosis such as cardiovascular parameters and hereditary thrombophilia, respectively, are prevalent also in MPN patients and appear to mediate additive prothrombotic propensity [17]. In addition, multiple MPN-specific factors promoting thrombosis have been identified recently. They comprise both quantitative and qualitative alterations of cellular blood components including erythrocytes, leukocytes, and platelets and extend to functional changes of the endothelium and the hemostatic cascade, thus mediating an overall hypercoagulable state. It has also been established that the presence of the* JAK2*V617F mutation associates with increased thrombotic potential, whereas the role of the actual* JAK2*V617F allele burden remains controversial. The recently developed international prognostic score for risk of thrombosis in ET (IPSET-thrombosis) is implementing advanced age, previous thrombosis,* JAK2*V617F, and cardiovascular risk factors [25]. Although not considered by the IPSET scoring, leukocytosis and increased hematocrit represent recognized and relevant promoting factors of thrombosis acting mainly via increased blood viscosity, platelet activation through leukocyte-platelet interactions, and secretion of microparticles of activated leukocytes [60]. Elevated platelet count has not been validated as an independent contributing factor, but functional changes have been observed. MPN platelets show increased biosynthesis of thromboxane A2 [61] and the circulating platelet pool in PV and ET is enriched in immature platelets which circulate in an activated state with increased expression of P-selectin and tissue factor [61, 62]. It has been found that platelets in PV and ET, particularly if carrying the* JAK2*V617F mutation, show increased potential of thrombin generation [63, 64]. Of note, platelet activation products such as PF4 are increased in plasma of MPN patients [63], which could facilitate an anti-PF4 immune response based on increased abundance of antigen. This process could be even more pronounced in patients not on low dose aspirin, which very efficiently reduces thromboxane A2 biosynthesis already at low doses and counteracts platelet activation [61]. Other factors like platelet-leukocyte aggregates, increased production of inflammatory cytokines and reactive oxygen species (ROS), and increased circulating endothelial cells as well as lower levels of protein C illustrate the broad variety of factors implicated in thrombosis in MPN [17]. Functional studies will be needed to determine the significance of their contribution to overt thrombosis in MPN patients. In addition, the broad range of proaggregatory and procoagulatory factors promoting thrombosis formation in MPN is probably not exclusive and additional, yet unidentified factors may further contribute to the complex pathogenesis of multifactorial thromboses in MPN.

Anti-PF4/heparin antibodies as seen in HIT could represent an additional prothrombotic factor in MPN. Several case reports of HIT occurring upon heparin treatment in PV and ET have been published suggesting that patients with MPN might be at risk for HIT-related immune responses and potentially thrombotic events [41, 42, 54]. HIT is a severe, prothrombotic condition facilitating arterial or venous thromboses triggered by antibody formation against PF4/heparin complexes upon treatment with heparin [31]. Diagnostic criteria of HIT include onset of thrombocytopenia within 5–14 days of heparin exposure along with detection of platelet-activating anti-PF4/heparin antibodies, while overt thrombosis is seen in up to 50% of cases [65]. However, these diagnostic criteria are hampered in PV and ET by baseline thrombocytosis which may mask thrombocytopenia and by the fact that thrombosis in MPN is obviously multifactorial [41, 42]. Insight into the pathogenesis of HIT has been gained which could provide links to a role of anti-PF4/heparin antibodies in PV and ET. Crystallization studies have shown that negatively charged heparin is complexing with tetramers of the cationic platelet factor 4 (PF4) after release from activated platelets. Linearization of heparin molecules allows multiple PF4 tetramers to bind, building substantial amounts of PF4/heparin antigen with neoepitopes on PF4 emerging under specific conditions [66]. Formation of anti-PF4/heparin antibodies is induced probably via recognition of PF4/heparin complexes by toll-like receptors [67], and PF4/heparin antibody complexes mediate activation of platelets via binding to the surface Fc*γ*RIIA receptor. Activation of monocytes via binding of PF4/heparin complexes to Fc*γ*RIIA and Fc*γ*RI enhances thrombin formation by increased tissue factor expression, which further promotes antibody formation by increasing platelet activation and PF4 levels [68, 69]. Fc*γ*RIIA and Fc*γ*RIIIA are involved in clearance of activated platelets mediating platelet depletion in HIT. Polymorphism in exon 4 of the Fc*γ*RIIA, H131R (c519G>A; rs1801274) associated with a histidine to arginine substitution, is known to infer a higher risk for overt thrombosis in patients with HIT homozygous for the 131RR allele due to increased platelet activation and tissue factor expression [69]. Also, Fc*γ*RIIA 131RR homozygous patients show reduced inhibitory effects by normal immunoglobulins of IgG2 subtype on Fc*γ*RIIA due to decreased affinity of Fc*γ*RIIA 131RR for IgG2 [69]. In addition to platelet activation via binding of anti-PF4/heparin antibodies to Fc*γ*RIIA via their Fc part, anti-PF4/heparin antibodies have also been found to bind PF4 complexed with endogenous GAGs on platelet, monocyte, and endothelial cell surfaces via their Fab domains [67].

From studies so far, it is evident that heparin represents the major trigger of the anti-PF4 immune response in HIT and shows differential immunogenicity for distinct types of heparin. Exposure to unfractionated heparin (UFH) provokes HIT in 0.3–3% [32], while frequencies are lower at 0.2–0.8% on low molecular weight heparin [70]. A study on minimized heparin use showed reductions in anti-PF4/heparin antibody positivity and clinical HIT in hospitalized patients, which highlights heparin exposure as the central inducing factor [71]. Additional factors supporting the development of anti-PF4/heparin antibodies and HIT have been described. Upon heparin exposure, trauma and surgery were associated with increased risk for HIT as compared to general internal medicine patients. This higher propensity for anti-PF4/heparin immune responses in surgical patients was related to tissue damage which could mediate release of glycosaminoglycans (GAG) as endogenous heparin-like polysaccharides and promote platelet activation with higher levels of PF4 in plasma [31] facilitating anti-PF4/heparin antibody formation due to increased availability of antigen. Inflammatory stimuli related to surgical tissue damage could further enhance the immune response to PF4/heparin complexes [37, 38]. This concept was supported by increased IL6 levels in patients after cardiovascular surgery with high-titer anti-PF4/heparin antibodies [72]. Also, the inflammatory milieu in the setting of bacterial infections has been shown to enhance anti-PF4/heparin antibody formation and HIT [35, 36]. PF4/heparin antibody formation may also be facilitated in other conditions characterized by elevated plasmatic PF4 levels such as diabetes [73], atherosclerosis [74], and cardiovascular [75] and renal disease [76]. A study on diabetic patients with increased vascular risk found anti-PF4/heparin antibodies more frequently than in nondiabetic patients [77]. Similar findings have been reported for patients with acute coronary syndrome (ACS) [78, 79] or cardiac surgery. Of note, positivity for anti-PF4/heparin antibodies has been associated with increased complication rate and mortality in cardiovascular patients [80].

Interestingly, one study suggested that inflammatory stimuli and platelet activation due to tissue injury upon joint replacement surgery would suffice to evoke anti-PF4/heparin antibodies in the absence of heparin treatment [39, 40]. Anti-PF4/heparin antibodies were detected in 6.5% of patients with arthroplasty in the absence of heparin and in 15% when dynamic compression as thromboprophylaxis was applied. We hypothesized that a similar constellation could be at play in a nonsurgical setting in patients with PV and ET. Platelet production and turnover is increased in PV and ET patients and it is known that MPN platelets are circulating in an activated state and show prolonged activation as compared to normal platelets. As a consequence, plasma levels of PF4 are increased [17]. Higher abundance of PF4 antigen and platelet activation could increase the susceptibility for anti-PF4/heparin antibody formation in MPN in the absence of heparin treatment. In addition, it is established that MPN create an inflammatory milieu with increased plasma levels of multiple cytokines including IL6 [81]. Detailed in vivo studies have revealed that excessive inflammatory cytokines in MPN originate from both the malignant clone and nonmutant cells [82]. Substantial disease burden in MPN is attributed to inflammatory symptoms including fatigue, pruritus, and bone and muscle pain. Treatment with the JAK1/2 inhibitor ruxolitinib reduces cytokine levels and inflammatory symptoms in most MPN patients highlighting a relevance of the inflammatory milieu for disease burden [83]. The inflammatory condition in MPN could further increase the probability for anti-PF4/heparin antibody formation in the absence of heparin exposure similarly to the findings in nonheparinized surgical patients [40], thereby increasing the risk of overt HIT in PV and ET.

Evidence of anti-PF4/heparin immune responses in MPN is scarce so far. A limited number of case series and reports of single cases describe the occurrence of HIT in PV and ET, while cases in MF have not been reported to date (Table 1) [41, 42, 54]. It is challenging to determine the significance of HIT for thrombosis in PV and ET based on this limited data. Furthermore, diagnostic work-ups vary among different reports. The presence of anti-PF4/heparin antibodies has not been assessed in all patients and antibody isotype testing was performed in a minority. Therefore, we investigated a large cohort of 127 MPN patients for anti-PF4/heparin antibodies using a systematic diagnostic approach. As no cases of HIT have been reported so far in MF, we focused our analysis on 76 patients with PV and 51 with ET. We applied a 2-step algorithm using first an anti-PF4/heparin antibody screening ELISA for analysis of patients' plasma followed by confirmatory testing. As differential effects of immunoglobulin isotypes for platelet activation and thrombin generation in HIT are known, positive patients were subjected to immunoglobulin isotype testing with a specific anti-PF4/heparin antibody ELISA determining positivity for IgG, IgM, and IgA, respectively. This uniform work-up in a large cohort of MPN patients for the first time provides consistent data on the frequency of anti-PF4/heparin antibody formation in PV and ET.

Antibody screening detected anti-PF4/heparin immunoglobulins in 17.3% of all MPN patients in the absence of heparin exposure (Table 4). Antibody formation was more frequent in PV than in ET with 21% and 11.8% of patients, respectively. These findings are notable, as anti-PF4/heparin antibodies in PV and ET patients without direct heparin exposure occur at a clearly higher frequency than in patients on heparin treatment known to develop antibodies in 0.3–3% [32]. Anti-PF4/heparin antibodies were also substantially more prevalent in PV and ET than in healthy individuals, in whom detection of anti-PF4/heparin antibodies is rare [84]. Subsequent validation testing confirmed anti-PF4/heparin antibodies in 14.5% of PV and 9.8% of ET patients which consolidates that anti-PF4/heparin antibody formation in MPN exceeds the risk in heparin-treated patients (Table 4, Figure 1). Also, anti-PF4/heparin antibody production was sustained as documented in a subset of PV and ET patients with serial testing at average intervals of 568 days for anti-PF4/heparin IgG and 660 days for IgM isotypes. A similar susceptibility for anti-PF4/heparin immune responses has been reported in nonheparinized surgical patients with dynamic compression therapy which lends strong support to our findings of prevalent anti-PF4/heparin antibodies independent of direct heparin exposure in nonsurgical, but hematological patients. Although there is no direct tissue damage in PV and ET, increased platelet activation and PF4 plasma levels are characteristic [17] and an inflammatory milieu is prominently present, as shown by clinical trials and experimental animal models [82, 83].

For further characterization of the anti-PF4/heparin immune response in MPN, we performed immunoglobulin isotype testing in PV and ET to delineate the proportion of anti-PF4/heparin IgG with an established role in platelet activation via Fc*γ*RIIA [31]. Anti-PF4/heparin IgG were detected in 9.2% of PV patients which exceeds the frequencies seen in heparin-exposed patients [32] or healthy individuals [84] (Table 4, Figure 1). Interestingly, anti-PF4/heparin antibodies in ET patients were of IgM isotype for which a role in pathogenesis of HIT is controversial. As case reports of overt HIT in ET have been published [41, 42, 46–51], but exclusively IgM are detected in our ET cohort, functional effects of IgM should be evaluated in future studies. Isotype specification of anti-PF4/heparin antibodies in our cohort did not identify any IgA-positive patients and none of the patients tested positive for multiple isotypes, whereas studies on the dynamics of anti-PF4/heparin immune responses have reported simultaneous detection of IgG, IgM, and IgA [33]. These differences in antibody isotype pattern may relate to differential immune mechanisms involved in anti-PF4/heparin immune responses in patients on heparin treatment versus patients with PV and ET.

Correlation with clinical characteristics revealed a tendency for more thrombotic events in PV patients with anti-PF4/heparin IgG immunoglobulins. We observed a 31% increase of relative risk for thrombotic complications in IgG-positive PV patients as compared to IgG-negative PV suggesting that anti-PF4/heparin IgG antibodies could potentially contribute to the multifactorial thromboses occurring in PV. Higher patient numbers would have been required to reach statistical significance of this finding, but the rarity of MPN is impeding the formation of more extensive patient cohorts (Tables 5 and 6). The fact that anti-PF4/heparin antibodies in PV patients with thrombosis were of IgG isotype may support a functional relevance of anti-PF4/heparin immune responses for increased thrombotic risk. Collaborative multicenter efforts will be required to further extend patient numbers even in rare disorders like MPN, to specifically explore the effect of anti-PF4/heparin IgG for overt thrombosis in PV and ET.

Special forms of HIT including spontaneous or delayed onset HIT are being discussed in rare cases. State-of-the-art diagnostic criteria for spontaneous HIT have been proposed and include thrombocytopenia and thrombosis without previous heparin exposure along with detectable anti-PF4/heparin antibodies with platelet-activating potential in the absence of heparin [85]. Delayed onset HIT may correspondingly occur days to weeks after heparin exposure. However, diagnostic criteria for these particular HIT phenotypes do not correspond well to the situation in PV and ET. Thrombocytopenia is often masked by excessive platelet production and pathogenesis of thromboses is strongly multifactorial. In MPN, it is the finding of surprisingly frequent, endogenous anti-PF4/heparin antibody formation including particularly IgG isotypes in PV in the current study which deserves attention. It should increase our awareness for platelet activation and inflammatory stimuli as contributors to the anti-PF4/heparin immune response in nonsurgical patients even in the absence of heparin exposure. Future collaborative multicenter studies will hopefully allow for analyses of more extensive patient cohorts also in these rare disorders to unequivocally determine the significance of anti-PF4/heparin antibodies for thrombotic risk in MPN. Functional studies will be required to characterize the mechanisms of the anti-PF4/heparin immune response in the absence of heparin exposure. Further insight into the functional basis of this phenomenon could inform measures to reduce anti-PF4/heparin antibody formation and potential thromboses. Our data demonstrate that PV patients need to be tested for anti-PF4 IgG at the latest when an actual thrombotic event occurs. As anti-PF4 IgG are endogenously prevalent at a substantial frequency in PV, their presence needs to be excluded before treatment with heparin, which could enhance anti-PF4/heparin immune response and thrombotic risk [55], is initiated to facilitate alternative anticoagulation in anti-PF4 IgG-positive PV patients. Prospective studies of anti-PF4/heparin antibody formation in MPN should follow and will lead to more general recommendations for diagnostic and clinical management of PV and ET patients with anti-PF4/heparin antibodies.

## Figures and Tables

**Table 1:** Reports of heparin-induced thrombocytopenia in myeloproliferative neoplasms. Three case series with more than one patient and nine single cases of heparin-induced thrombocytopenia (HIT) in polycythemia vera (PV) and essential thrombocythemia (ET) have been reported. Number of patients, manifestation of thrombosis, and diagnostic work-up of anti-PF4/heparin antibodies are indicated. PE: pulmonary embolism, DVT: deep vein thrombosis, SVT: sinusoidal vein thrombosis, TIA: transitory ischemic attack, PF4: platelet factor 4, Ab: antibody, ELISA: enzyme linked immunosorbent assay, PAT: platelet aggregation testing, HIPA: heparin-induced platelet aggregation, SRA: serotonin release assay, PaGIA: platelet factor 4/heparin-particle gel immunoassay, and HemosIL-HIT: automated rapid testing for platelet factor 4/heparin antibodies.

	Report	MPN type	Patients (*n*)	Thrombosis type (*n*)	PF4/heparin Ab testing (*n*)	Test type	Ab isotype testing
Case series	Bovet et al., 2016	ET	2	Stroke (2)	2	ELISA, PAT	−
	Randi et al., 2010	PV	2	PE (2)	1	ELISA, HIPA	+
		ET	3	PE (3)	1		+
	Spectre et al., 2008	PV	2	Catheter-associated (1), PE (1), skin necrosis (1)	2	PaGIA	−
		ET	1		1		−

Single cases	Biagioni et al., 2013	PV	1	Budd-Chiari (1)	1	HemosIL-HIT	−
	Akoum et al., 2009	PV	1	Budd-Chiari (1)	1	ELISA	−
	Hayashi et al., 2004	PV	1	PE	1	ELISA	−
	Garcia et al., 1991	PV	1	DVT, PE (1)	1	PAT	−
	Kyritsis et al., 1990	PV	1	Thrombophlebitis (1)	1	HIPA, SRA	−
	Murawaki et al., 2012	ET	1	Stroke (1)	1	ELISA	−
	Richard et al., 2011	ET	1	SVT (1)	1	ELISA	+
	Lapecorella et al., 2010	ET	1	DVT, PE (1)	1	ELISA	−
	Houston, 2000	ET	1	Axillary DVT (1)	1	SRA	−
	Risch et al., 2000	ET	1	TIA (1)	1	ELISA	−
	Walther et al., 1996	ET	1	SVT (1)	—	—	−

**Table 2:** Baseline characteristics of MPN patient cohort. Characteristic parameters of the study population of 127 MPN patients are displayed (mean for age and peripheral blood counts, frequency for all other parameters). Frequencies are indicated in percent of total patients: absolute numbers are given in parentheses.

MPN type	Polycythemia vera	Essential thrombocythemia
*Patient cohort*	total *n* = 127	
Patients (*n*)	76	51
Male (*n*)	42	18
Female (*n*)	34	33
*Parameters at diagnosis*		
Age (*y*)	55.8	53.2
Hematocrit (%)	53.2	41.6
Hemoglobin (g/l)	176.2	139.2
Platelets (G/l)	602.9	984.3
Leukocytes (G/l)	12.4	9.0
Splenomegaly (%)	55.3 (42/76)	41.2 (21/51)
*JAK2*V617F (%)	84.2 (64/76)	52.9 (27/51)
*Thrombohemorrhagic events*		
Thrombosis	44.7 (34/76)	39.2 (20/51)
Hemorrhage	18.4 (14/76)	3.9 (2/51)
*Medications*		
Antiaggregation	94.7 (72/76)	88.2 (45/51)
Oral anticoagulation	30.3 (23/76)	11.8 (6/51)
Cytoreduction	75.0 (57/76)	68.6 (35/51)

**Table 3:** Site of thrombotic events in MPN. The site of thromboses in 127 MPN patients is displayed highlighting the relevance of thrombotic complications for disease burden in MPN. CNS: central nervous system, VTE: venous thromboembolism, DVT: deep vein thrombosis, PE: pulmonary embolism, and PAD: peripheral arterial disease.

Site of thrombosis	Frequency	
	(%)	*n*
Total	100	54
CNS	37.0	20
VTE		
DVT	20.4	11
PE	11.1	6
Cardiac	14.8	8
Splanchnic	13.0	7
PAD	3.7	2

**Table 4:** Characterization of anti-PF4/heparin antibody formation in MPN. A cohort of 127 patients with myeloproliferative neoplasms (MPN) including polycythemia vera (PV, *n* = 76) and essential thrombocythemia (ET, *n* = 51) were characterized for anti-PF4/heparin antibodies. Analysis by a screening ELISA was subsequently validated by follow-up testing and antibody isotype testing determining IgG, IgM, and IgA. PF4: platelet factor 4; Ig: immunoglobulin.

MPN		PF4/heparin antibody characterization				
Subtype	Patients (*n*)	Screening (% positive)	Validation (% positive)	Isotype testing (% positive)		
				IgG	IgM	IgA
PV	76	21.1 (16/76)	14.5 (11/76)	9.2 (7/76)	5.3 (4/76)	0.0 (0/76)
ET	51	11.8 (6/51)	9.8 (5/51)	0.0 (0/51)	9.8 (5/51)	0.0 (0/51)

Total	127					

**Table 5:** Impact of anti-PF4/heparin antibodies on thrombotic risk in polycythemia vera. Anti-PF4/heparin antibodies of IgG isotypes, which have a known implication in pathogenesis of HIT, were assessed for a potential impact on thrombotic risk in polycythemia vera. IgG positivity conferred a 31% increase of relative risk for thrombosis as compared to IgG-negative PV. Statistical significance was not reached due to sample number. PF4: platelet factor 4; Ig: immunoglobulin.

	Anti-PF4/heparin IgG isotype		Relative thrombotic risk	*p* value
	Positive	Negative		
Thrombotic complications (%)	57.1	43.5	1.31	>0.05
No thrombotic complications (%)	42.9	56.5		

**Table 6:** Thromboembolic complications in anti-PF4/heparin IgG-positive PV patients. Thromboembolic complications are indicated in 7 PV patients positive for anti-PF4/heparin antibodies of IgG isotype. Thromboembolic events occurred in patients 1–4 and not in patients 5–7. Both arterial and venous complications as well as repeated events were seen. Splenic infarcts as in patient 5 are not considered classic thromboembolic complications in MPN. Grade IV PAD induced critical ischemia in patient 6 without an acute occlusive event. Pat: patient, Dgn: diagnosis, DVT: deep vein thrombosis, TIA: transitory ischemic attack, CVI: cerebrovascular infarction, NIHSS: National Institute of Health Stroke Scale, PE: pulmonary embolism, CHD: coronary heart disease, and PAD: peripheral arterial disease.

Pat	Age at Dgn	Sex	MPN type	PF4/heparin Ab isotype	Thromboembolic complications (*n*)	1st event	2nd event
1	71	m	PV	IgG	2	2-level DVT with postthrombotic syndrome	TIA
2	49	m	PV	IgG	2	CVI (NIHSS 5 pt)	CVI (NIHSS 16 pt)
3	57	m	PV	IgG	1	Bilateral PE with pulmonal-arterial hypertension	—
4	33	m	PV	IgG	1	In-stent thrombosis in early onset CHD	—
5	41	m	PV	IgG	0	Splenic infarcts, no splenic vein thrombosis	—
6	61	m	PV	IgG	0	Critical ischemia of lower extremity in grade IV PAD	—
7	69	f	PV	IgG	0	—	—
